# Experimental and DFT Study of the Monocomponent and Binary Adsorption of Heavy Metals in Chitosan Hydrogels at Different Temperatures

**DOI:** 10.3390/polym18182247

**Published:** 2026-09-15

**Authors:** Billy Alberto Ávila Camacho, Norma Aurea Rangel Vázquez, Edgar A. Márquez Brazón, Verónica Janeth Landín Sandoval

**Affiliations:** 1 TecNM/Instituto Tecnológico de Aguascalientes, Aguascalientes 20256, Mexico; 2 Grupo de Investigaciones en Química y Biología, Departamento de Química y Biología, Facultad de Ciencias Básicas, Universidad del Norte, Carrera 51B, Km 5, vía Puerto Colombia, Barranquilla 081007, Colombia; 3 Departamento de Nanotecnología, Universidad Tecnológica Metropolitana de Aguascalientes, Gerónimo de La Cueva s/n, Aguascalientes 20126, Mexico

**Keywords:** adsorbent, chitosan, heavy metals, multicomponent adsorption

## Abstract

High levels of global water pollution have driven the development of effective strategies for removing priority contaminants; this is a worldwide issue that calls for innovation in traditional methodologies using affordable materials with high removal capacities. Experimental and DFT studies were combined to elucidate the adsorption, selectivity, and competitive mechanisms of Hg^2+^, Ni^2+^, and Cu^2+^ onto a chitosan-based hydrogel, linking experimental behavior with molecular-level interactions. FTIR and XRD analysis allowed the determination of the hydrogel composition, the verification of the cross-linking of the polymer with the cross-linking agent, and the detection of heavy metals analyzed on the adsorbent. After the characterization, the isotherms were experimentally obtained in single and binary systems at 298.15, 303.15, and 313.15 K and pH 4. In binary experiments, antagonistic adsorption occurred due to competitive binding of heavy metals to the hydrogel’s active sites. The highest adsorption capacities achieved from individual solutions were 0.723, 0.187, and 0.420 mmol/g at 313.15 K for Hg^2+^, Ni^2+^, and Cu^2+^, respectively, and those for binary solutions were 0.633, 0.080, and 0.238 mmol/g at 313.15 K for Hg^2+^, Ni^2+^, and Cu^2+^, respectively. Adsorption kinetics in both single- and binary systems were well fitted by a pseudo-second-order model, consistent with a chemisorption-controlled mechanism. The adsorption process was endothermic and spontaneous, and it was determined that the adsorption was attributed to primary amine and hydroxyl groups available on the surfaces of the cross-linked hydrogels. DFT calculations revealed preferential interactions of Hg^2+^, Ni^2+^, and Cu^2+^ with specific hydrogel functional groups, supporting the experimental adsorption selectivity.

## 1. Introduction

Water is essential for flora, fauna, and human life; however, the quality of water resources is rapidly deteriorating due to the presence of heavy metals, dyes, pesticides, and residues from pharmaceuticals and personal care products caused by human and industrial activities [1,2]. Heavy metals posing a serious threat to aquifers include Al, Ni, Cu, Fe, Mn, Cd, Hg, Pb, As, and Cr [3,4]. In water, chromium is found as Cr (III) and Cr (VI), but the Cr (VI) is the more toxic form; at high concentrations, it causes vomiting, cramps, convulsions, death, gastrointestinal pain, lung cancer, and dermatitis, whereas nickel causes cancer, lung problems, or kidney failure. Finally, mercury damages the central nervous system [5].

Currently, various processes exist for removing heavy metals from water, such as adsorption [6], chemical precipitation [7], nanofiltration [8,9], flocculation [10], and ion exchange [4]. Nevertheless, adsorption stands out as an effective, economical, and suitable process for removing contaminants from water [11,12]. Prominent adsorbent materials include hydrogels, zeolites, activated carbon, and clays. However, hydrogels possess a greater number of active sites for contaminant removal due to their three-dimensional structure composed of polymers like chitosan, which can absorb and retain water, thereby increasing in volume [13,14]. The structure of the hydrogels allows for an adsorption capacity ranging from 2.3 to 2240 mg/g [15].

The structure of chitosan contains N-acetyl-D-glucosamine and D-glucosamine units linked by a glycosidic bond [16,17,18,19]. Chitosan possesses various properties, such as biocompatibility and antibacterial and antiviral activity; furthermore, it is non-toxic, non-allergenic, capable of forming films, and exhibits high elasticity [20]. The use of chitosan as an adsorbent has been investigated due to its cationic nature; its molecular chain encapsulates suspended solid particles, minerals, and heavy metals such as zinc, chromium, arsenic, titanium dioxide, copper, nickel, phosphorus, and especially mercury, lead, and uranium, as well as dyes, amino acids, and proteins [21,22,23,24,25]. Specifically, the cross-linking between chitosan and glutaraldehyde occurs via a Schiff condensation reaction, in which amine functional groups react with aldehyde groups. Consequently, this reaction reduces the number of amine groups while forming various imine groups [26].

Chitosan-based hydrogels possess a three-dimensional porous architecture that enhances the diffusion of contaminants through the polymer matrix, increases the exposure of functional groups, and promotes stronger interactions between the adsorbate and the adsorbent, as well as higher adsorption efficiency compared to chitosan flakes [27]. Accordingly, during the adsorption of heavy metal ions onto chitosan-based hydrogels, multiple mechanisms influencing overall adsorption efficiency can occur simultaneously, such as π-π interactions, hydrogen bonding, and metal complex formation. However, surface complex formation is the most significant mechanism, as it plays a predominant role in the adsorption of heavy metal ions [28]. Surface complexation depends on the interaction between surface functional groups and heavy metal ions. Positively or negatively charged groups present on the chitosan surface—such as hydroxyl, carboxyl, or amino groups—attract oppositely charged ions in aqueous solution and form complexes via chemical bonds at the surface.

Although some functional groups are lost during the cross-linking process to form the three-dimensional matrix, many remain free and exposed to the hydrogel surface, allowing them to donate their electron pairs to coordinate and chelate metal cations [29]. Studying the adsorption process of metals such as Ni^2+^, Cu^2+^, and Hg^2+^ onto chitosan-based hydrogels is crucial for developing materials capable of removing contaminants from water, as chitosan presents active sites that facilitate interaction with adsorbates through various chemical mechanisms.

Barbosa et al. [30] synthesized chitosan-based hydrogels using acetic acid and glutaraldehyde, where the hydrogel with glutaraldehyde has water adsorption superior to 300%, representing an excellent material for copper and chromium removal. Stanković et al. [31] evaluated a chitosan/cellulose/glutaraldehyde hydrogel for copper removal in batch and dynamic systems; the hydrogel demonstrated excellent stability and reusability after multiple cycles, and increasing the height of the fixed-bed column extended the exhaustion times, leading the researchers to conclude that the hydrogel was efficient for copper adsorption. Aljar et al. [32] studied the adsorption of lead, cadmium, zinc, and cobalt using chitosan/PVA/cellulose spheres; using SEM, they determined that the spheres retained the spherical shape before and after adsorption and exhibited an efficiency of 84% to 99% at 60 min, with efficiency remaining above 80% even after five adsorption-desorption cycles. Vieira et al. [33] used X-ray photoelectron spectroscopy (XPS) to study the removal of copper, mercury, and chromium using chitosan hydrogels cross-linked with glutaraldehyde and epichlorohydrin; their results indicated that functional groups can give rise to different adsorption mechanisms, as the metals bind to the chitosan/glutaraldehyde system differently than to the chitosan/epichlorohydrin system, and they determined that the glutaraldehyde-cross-linked hydrogels exhibited a higher adsorption capacity for mercury.

The mechanisms of adsorption can be understood at the atomic level using simulations based on density functional theory (DFT), which allow for the identification of adsorption energies, preferential binding sites, and the physical, chemical, and biological properties of molecules or macromolecules. Simulations of binary and multicomponent heavy metal systems provide information on competition and selectivity among the metals. Once obtained, this information can be used to optimize process efficiency in real-world applications.

DFT simulations show that the hydroxyl and amino groups present in chitosan provide electron pairs that can be readily donated to heavy metal ions—such as copper, mercury, nickel, zinc, and lead—resulting in the formation of a stable metal complex [34]. Vieira et al. [35] used DFT to determine the adsorption capacity for Pb (II) and Cu (II) using N-lauroyl chitosan. Furthermore, the functional groups enhanced molecular interactions between the ion and the adsorbent. Ezzat et al. [36] achieved improved selectivity in the adsorption of heavy metals (Pb, Cd, As, Cu, Ni, and Fe) onto chitosan by employing DFT. Mardani et al. [37] analyzed chitosan-based compounds and determined the maximum adsorption of Cu^2+^ and Ni^2+^ ions. While Cu^2+^ adsorption onto chitosan-based hydrogels has been extensively investigated, studies involving Hg^2+^ and Ni^2+^ are comparatively limited, especially in binary systems, where competitive adsorption behavior and mechanistic insights remain poorly understood.

Bansal et al. [38] analyzed the lead and mercury adsorption capacity of chitosan hydrogels during the 2020–2025 period using DFT (Density Functional Theory), isotherms, and kinetics, proposing the use of multifunctional adsorbents and their evaluation in real water. Then, the main objective of this article was the preparation of chitosan-based hydrogels to obtain different adsorption isotherms of Hg^2+^, Cu^2+^, and Ni^2+^ in single and binary systems. The material was characterization using different techniques such as FTIR, XRD, and SEM, to analyze the chemical composition of the adsorbent, its crystalline structure, and the morphological structure of the material.

## 2. Materials and Methods

### 2.1. Materials

The materials used in this study, including chitosan (low molecular weight 50,000–190,000 Da), mercuric chloride (HgCl_2_), copper (II) nitrate (Cu(NO_3_)_2_·6H_2_O), nickel (II) nitrate (Ni(NO_3_)_2_·6H_2_O), and a glutaraldehyde solution (25% purity), were purchased from Sigma-Aldrich (Sigma-Aldrich Química S. de RL. de C.V, 50200, Toluca, Mexico). Additionally, acetic acid (99.7% purity) was obtained from J. T. Baker and deionized water (DI water) was supplied by MERCK (MERCK Mexico, 53500, Naucalpan de Juárez, Mexico).

### 2.2. Synthesis of Chitosan Hydrogels

1.5 g of chitosan powder was dissolved in 100 mL of 3% *v*/*v* acetic acid solution. Then, the mixture was stirred using a magnetic hotplate at 80 RPM for 3 h at a temperature of 50 °C. Subsequently, 1% *v*/*v* glutaraldehyde solution was slowly added, and the solution was stirred for an extra 2 h. The resulting mixture after 5 h of stirring was pale yellow in color and had a viscous consistency. Subsequently, the solution was centrifuged at 6000 rpm for 5 min to sediment the suspended particles. Finally, 25 mL of the mixture was poured into different petri dishes and allowed to dry at room temperature for 5 days, which allowed the formation of glutaraldehyde-cross-linked chitosan films.

### 2.3. Characterization of Chitosan/Glutaraldehyde Hydrogel

The swelling experiments of glutaraldehyde-cross-linked chitosan were carried out in a batch system using deionized water at pH 4 for 2 h. The swelling rate was calculated using the Equation (1) [39].

(1) $$SR\;\%=\frac{W_{s}-W_{d}}{W_{d}}*100$$

where, $W_{s}$ represents the weight of the swollen hydrogel and $W_{d}$ indicates the weight of the dry hydrogel.

FTIR analysis was performed using a Thermo Scientific Nicolet iS10 spectrophotometer. In this analysis, 32 scans were performed, and the spectra were recorded in a range of wavenumbers from 4000 to 600 cm^−1^. To determine the crystallinity, an XRD analysis was performed using a Malvern Panalytical diffractometer equipped with a PIXcel ID detector. The equipment was operated at 45 kV and 40 mA and a wavelength of 1.5406 Å.

The surface of the hydrogel was analyzed using a Coxem EM-30 SEM equipped with a secondary electron detector (SED). The equipment was operated at 10 kV. The samples were cut to a suitable size, mounted on a sample holder and fixed with double-sided carbon adhesive tape. The samples were coated with a thin layer of gold to ensure conduction and to avoid electrostatic charging during the process. Finally, the samples used were observed under vacuum conditions using an electron detector.

### 2.4. Adsorption Studies of Hg^2+^, Ni^2+^, and Cu^2+^

500 mL of solution was prepared with 2 mmol/L of each of the salts (mercuric chloride, copper nitrate, and nickel nitrate) with pH 4. The adsorption experiments were deliberately conducted at pH = 4 to guarantee that the divalent metallic species (Hg^2+^, Ni^2+^, and Cu^2+^) remain predominantly as free, fully soluble aquo-cations, strictly preventing the chemical precipitation of insoluble metal hydroxides [M(OH)_2_] or hydroxy-complexes that typically take place under weakly acidic to alkaline conditions (pH 5.5–6). While higher removal efficiencies are frequently reported at alkaline pH, such apparent gains often arise from non-adsorptive hydroxide precipitation rather than genuine surface chelation.

Furthermore, pH = 4 provides an optimal compromise that minimizes excessive hydronium ion (H_3_O^+^) competition while closely mimicking the characteristic acidic conditions of real metal-bearing industrial streams, such as acid mine drainage and electroplating effluents (pH = 3–5) [7].

The concentration of the adsorbates was kept fixed while the mass of the hydrogel varied from 0.01 to 0.07 g. The experiments were run in batches using a volume of 10 mL at temperatures of 298.15, 303.15, and 313.15 K and stirring at 120 rpm for 24 h. Afterward, the adsorbent was separated from the solutions with the metals and diluted to be quantified by atomic absorption spectroscopy (iCE 3000 series spectrometer, Thermo Fisher Scientific Inc, León, México). All experiments were performed in duplicate. To obtain the adsorption capacities, Equation (2) was used [40].

(2) $$q_{e}=\frac{\left(Co-Ce\right)V}{m}$$

where $“q_{e}”$ is the adsorption capacity, “*Co*” and “*Ce*” are the initial and equilibrium concentrations of the adsorbate solutions, respectively, “*V*” is the volume of the solution used, and “m” is the mass of the hydrogel. To determine the thermodynamic parameters (Δ*G*°, ΔH°, and ΔS°), the apparent distribution coefficient (*Kd*) was first calculated using the following Equation (3) [41]:

(3) $$Kd=\frac{qe}{Ce}$$

The coefficient *Kd* was converted to a dimensionless constant by multiplying it by the density of water. Subsequently, the standard thermodynamic equilibrium constant (*K*°c) was obtained by extrapolating ln(*Kd*) to a concentration of zero and plotting it as a function of *Ce*. The values of ΔH° and ΔS° were calculated from the slope and intercept, respectively, by linearly fitting the van′t Hoff Equation (4) [42].

(4) $$\ln K{}^{\circ}c=\frac{∆S{}^{\circ}}{R}-\frac{∆H{}^{\circ}}{RT}$$

where T is the temperature (K) and R is the universal gas constant (8.31 J/K/mol). Finally, the standard Gibbs free energy change (∆G°) was determined using the following fundamental relationship (see Equation (5)) [42]:

(5) ∆G°=−RTlnK°c

For desorption, the metal-loaded hydrogel was rinsed with deionized water and stirred in a 0.1 M HNO_3_ stripping solution to elute the coordinated metallic cations via proton exchange. The regenerated adsorbent was subsequently washed with deionized water until neutral pH and reused in fresh metal solutions under identical operational conditions for four additional cycles. The reusability efficiency (RE%) for each cycle (*n* = 1–5) was calculated by applying Equation (6).

(6) $$RE\left(\%\right)=\frac{Q_{ads,n\;}}{Q_{ads,1}}\times 100$$

### 2.5. Modeling of Single-Component and Binary Adsorption Systems

#### 2.5.1. Kinetic Modeling

The kinetic behavior of the adsorption process was evaluated using the following models. The pseudo-first-order model is expressed as follows [42]:

(7) $$\ln\left(q_{e}+q_{t}\right)=\;\ln\left(q_{e}\right)-\;k_{1}t$$

where q_t_ (mmol/g) is the amount of adsorbate at time t (min), q_e_ (mmol/g) is the adsorption capacity at equilibrium, and k_1_ (min^−1^) is the rate constant of the pseudo-first-order model. The parameters were obtained from the slope and intercept of the linear plot of ln(q_e_ − q_t_) versus t. The pseudo-second-order model is expressed in its linearized form as follows [42]:

(8) $$\frac{t}{q_{t}}=\frac{1}{k_{2}q_{c}^{2}}+\;\frac{1}{q_{e}}$$

where k_2_ (g/mg min) is the rate constant of the pseudo-second-order model. The values of q_e_ and k_2_ were calculated from the slope and intercept of the plot of t/_qt_ versus t.

To investigate the mass transfer mechanism and the rate-controlling steps governing the uptake of Hg^2+^, Ni^2+^, and Cu^2+^ across single and binary systems, experimental kinetic data were analyzed using the Weber–Morris intraparticle diffusion equation:

(9) $$q_{t}=kt^{1/2}+C$$

where q_t_ (mmol/g) is the adsorption capacity at time t (min), *k* (mmol/g min^1/2^) represents the intraparticle diffusion rate constant, and C (mmol/g) is the boundary layer thickness constant.

Linear piecewise regression of $q_{t}$ versus $t^{1/2}$ was carried out over two defined temporal intervals: the initial mass transfer stage (t ≤ 40 min) and the progressive diffusion/saturation stage (40–100 min). The kinetic parameters (*k*_1_, *k*_2_, *C*_1_ and *C*_2_) and the corresponding determination coefficients (*R*^2^) were determined from the slopes and intercepts of each linear region.

#### 2.5.2. Adsorption Isotherm Modeling

The experimental data for the single-component adsorption systems were modeled using the Sips isotherm [43]:

(10) $$q_{e}=\frac{q_{max}{\left(K_{s}C_{e}\right)}^{n_{s}}}{1+{\left(K_{s}C_{e}\right)}^{n_{s}}}$$

where, q_max_ is adsorption capacity (mmol/g), *K*_s_ is constant affinity of Sips (L/mmol), C_e_ is equilibrium metal concentration (mmol/L) and *n*_s_ is Sips heterogeneity parameter. The extended Sips equation was used for the binary adsorption systems [43]:

(11) $$q_{e}=\frac{q_{max,1,2}{\left(K_{s1,2}C_{e1,2}\right)}^{n_{s},1,2}}{1+{\left(K_{s,1}C_{e,1}\right)}^{n_{s},1}+{\left(K_{s,2}C_{e,2}\right)}^{n_{s}2}}$$

where, *K*_s1,2_ represents the Sips affinity constant for metals 1 and 2, respectively, *C_e_*_1,2_ is equilibrium concentration of metals 1 and 2, respectively, *q*_max,1,2_ is maximum adsorption capacity for metals 1 and 2, respectively, and *n*_s,1,2_ is Sips heterogeneity parameter for metals 1 and 2, respectively.

### 2.6. DFT Analysis

The computational analysis was performed on a Dell-brand PC with an i7 processor and 16 GB of RAM. Ions and adsorptions were analyzed individually in the hydrogel using the Spartan software with DFT (Spartan’14), def2-TZVP in water, neutral charge. In addition, zero-point energy (ZPE) corrections, thermal corrections (enthalpy and entropy), and solvation effects were included [44,45].

The initial geometries of the hydrogel–heavy-metal system were constructed using tools from the Spartan ’14 software. The metal cations (Hg^2+^, Cu^2+^, and Ni^2+^) were systematically placed at typical coordination distances (approx. 2.0–2.5 Å) adjacent to the primary electron-donor Lewis-basic heteroatoms of the polymer matrix, specifically the nitrogen atoms of the amine groups (NH_2_), the oxygen atoms of the primary and secondary hydroxyl groups (OH at C-3 and C-6 positions), and adjacent bridging/cross-linking groups. Each constructed initial complex configuration was subsequently subjected to unconstrained geometry optimization to locate the true local stationary points on the potential energy surface. The configuration exhibiting the lowest total electronic energy/binding energy was selected as the representative coordination complex.

The compute option calculates the IR spectrum, charges and bond orders, orbitals and energies, QSAR, molecule and thermodynamics properties, and vibrational modes. The temperature was configured in the same “thermodynamics” section; the software can generate different thermodynamic values at different temperatures without needing to simulate the molecule for each temperature. For chitosan, four glucosamine units were analyzed, where one unit was acetylated to simulate a deacetylation degree of 75%.

FTIR spectrum, and properties such as Gibbs free energy change (kJ/mol), dipole moment (Debye), area (Å^2^) and volume (Å^3^) were calculated after the geometry optimization using Spartan’14 software. The surface option shows the electrostatic potential map (EPM), which provides a contour plot of a molecule, indicating the sites of highest and lowest electron density. The same procedure was used to calculate these properties in the adsorption of the ions in the hydrogel using Spartan software. The corresponding adsorption parameters were calculated according to the following:

ΔGads° = Gcomplex − (Ghydrogel + Gion)(12)

ΔHads° = Hcomplex − (Hhydrogel + Hion)(13)

## 3. Results and Discussions

### 3.1. Computational Analysis

As indicated in Table 1, the ΔGads° values confirm a spontaneous adsorption process, driven primarily by the presence of hydroxyl and amino groups. Furthermore, the ΔHads° values reveal an endothermic and energetically stable mechanism [46]. Increasing the temperature enhances the spontaneity of the system; this additional thermal energy boosts metal ion mobility and attenuates internal electrostatic repulsive forces, thereby accelerating the formation rate of the chitosan–metal complex [47,48]. The incorporation of heavy metal cations significantly alters both the steric and electronic properties of the hydrogel. The formation of coordination bonds between the functional groups of the polymer and the metal ions induces substantial charge transfer and elevates the dipole moment. Concurrently, these bonds act as anchoring points that restrict the mobility of the polymer chains. This structural loss of flexibility causes the absolute value of ΔGads° to approach zero in these systems [49].

**Figure 1:**
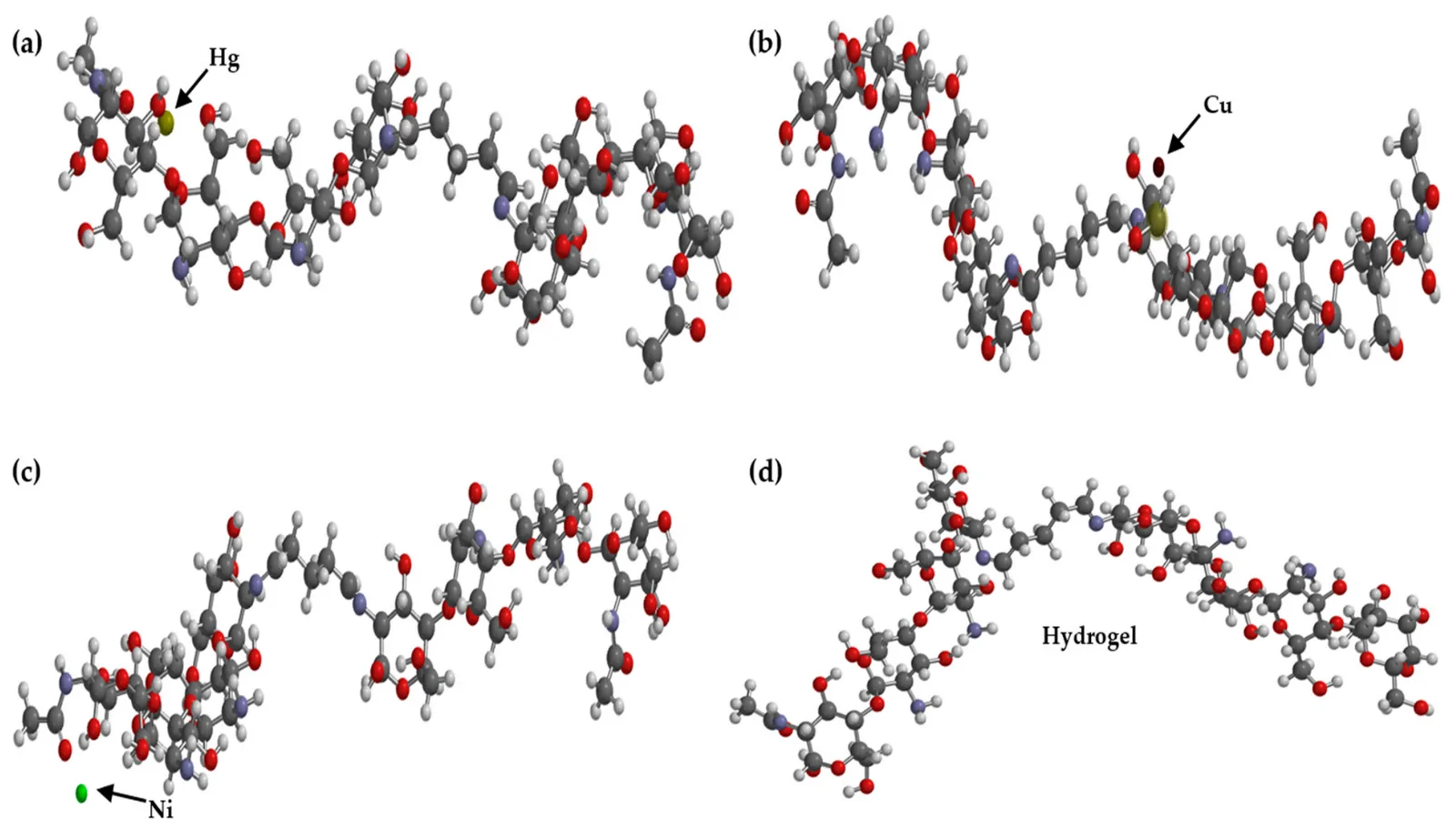
Geometries for optimizing the adsorption of (**a**) mercury, (**b**) copper, (**c**) nickel in chitosan hydrogel, and (**d**) chitosan-based hydrogel. Red color: oxygen, white color: hydrogen, yellow color: mercury, brown color: copper, blue color: nitrogen, gray color: carbon, and green color: nickel, respectively.

Among the evaluated metals, mercury adsorption exhibited the most negative ΔGads° values, reflecting a stronger thermodynamic driving force [50]. Consequently, the thermodynamic affinity sequence for the adsorption process follows the order Hg^2+^ > Cu^2+^ > Ni^2+^. This metal uptake occurs because chitosan functions as a robust chelating agent, synergistically supported by intermolecular interactions and hydrogen bonding [51,52,53]. Finally, the increased dipole moment corroborates the presence of strong adsorbent–adsorbate interactions, while heightened polarizability facilitates localized charge separation within the hydrogel, effectively drawing ions into the polymeric matrix (see Figure 1) [54].

**Figure 2:**
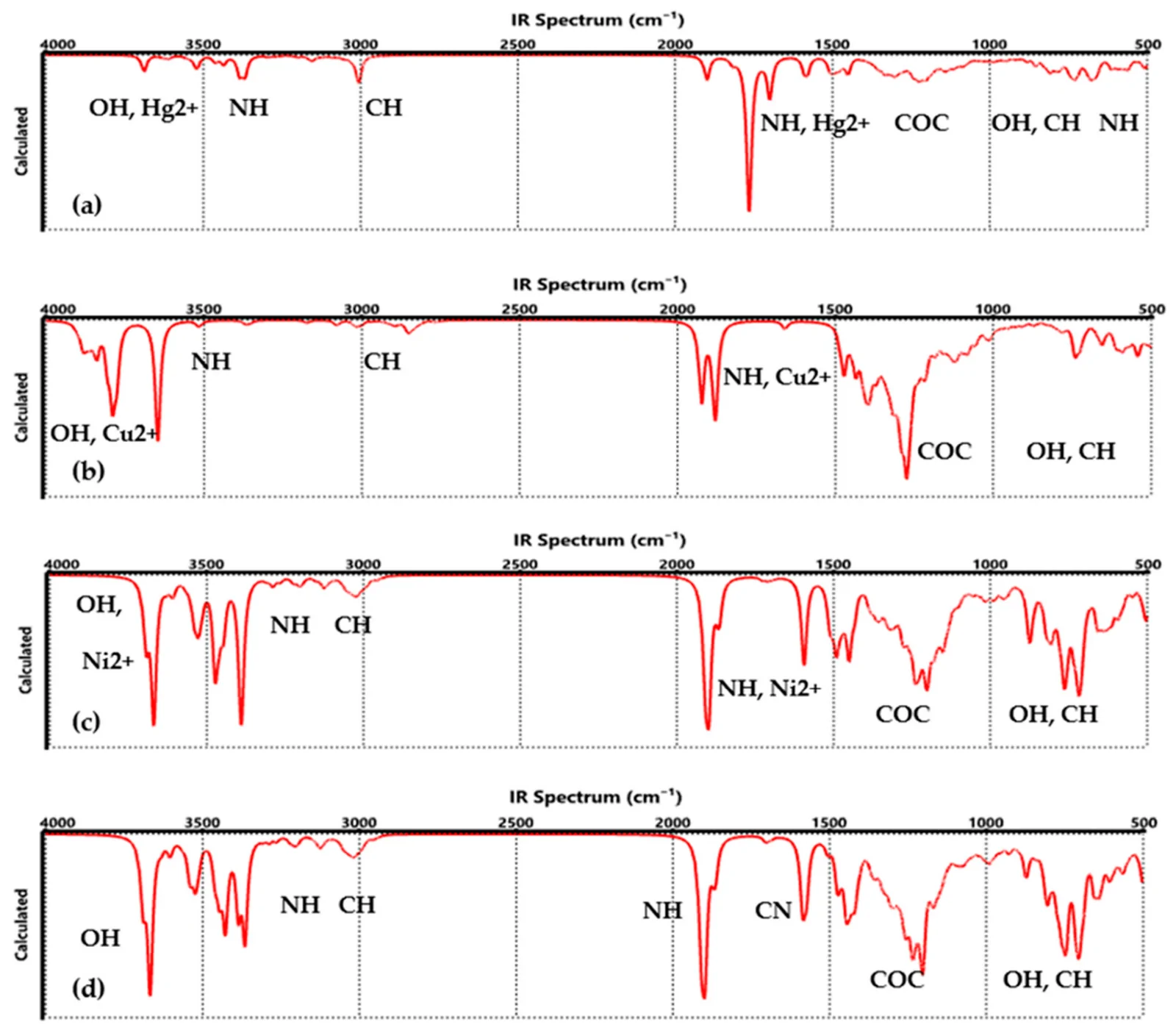
FTIR spectrum of the adsorption of (**a**) mercury, (**b**) copper, (**c**) nickel, and (**d**) chitosan based hydrogel.

The FTIR spectrum of mercury adsorption is shown in Figure 2a, where OH stretching occurred at 3688 and 3524 cm^−1^, the range from 3459 to 3384 cm^−1^ was attributed to the overlap of OH and NH stretching, and NH stretching was observed at 3163 cm^−1^ [55]. These values indicate that OH groups participate in the adsorption process [48]. CH vibrations were observed at 3163 and 3006 cm^−1^. In 1988 and 1765–1700 cm^−1^, the CN and CO stretching of the acetyl group were observed, while at 1590 cm^−1^, the adsorption of mercury was assigned and verified by vibrations at 1452 and 1345 cm^−1^, which indicated that glutaraldehyde could adsorb metal cations [46]. Additionally, the CH rocking vibration was observed at 1345 cm^−1^ [56]. At 1298–1207 cm^−1^, the CH, CC, and NH rocking vibrations were assigned. The region from 1137 to 1076 cm^−1^ was assigned to the OH, CC, and CH stretching of chitosan [53]. Finally, the OH vibrations were observed from 855 to 639 cm^−1^.

Figure 2b shows the FTIR spectrum of copper adsorption, where copper interactions with the OH stretch of chitosan were observed at 3851, 3826, and 3765 cm^−1^. Vibrations at 3517, 3357, and 1477 cm^−1^ corresponded to copper adsorption due to the OH, NH, and methoxy group stretches [51]. The aliphatic chain of chitosan was observed at 3175 cm^−1^ of the asymmetric CH stretch, while the symmetric CH stretch was observed at 3084, 3016, and 2846 cm^−1^ [47]. The C=O stretch of the acetyl group was determined in 1924 and 1879 cm^−1^. At 1323, 1274, 1230, and 1110 cm^−1^, CH and C-C stretching and NH rocking were observed [55]. At 777, 643, and 595 cm^−1^, NH and OH out-of-plane vibrations were attributed, and the Cu-O interaction was observed at 525, 477, and 441 cm^−1^.

The FTIR spectrum of nickel adsorption is shown in Figure 2c, where the peaks at 3818 and 3616 cm^−1^ corresponded to OH stretching [57] and interactions with nickel [57]. NH stretching was assigned at 3364 cm^−1^ [49,58] with a high-intensity peak, which can be attributed to the interaction with nickel [50]. The asymmetric and symmetric CH stretching were observed at 2779 and 2742 cm^−1^, and the peaks at 1923 and 1888 cm^−1^ were assigned to the C=O stretching of the acetyl group. The peak at 1630 cm^−1^ was attributed to the primary amines of chitosan [57,58]; the methyl group was located at 1473 cm^−1^, and the CH deformation of CH_2_ and OH stretching were assigned to 1428 and 1319 cm^−1^. The NH stretching and CH bending of CH_2_ were observed at 1380 and 1268 cm^−1^ [56], and the NH bending was determined at 1319 cm^−1^. The peak at 1131 cm^−1^ was attributed to the C-O [56,58] and CH (CH_2_) stretching of the chitosan structure [57]. The peak at 747 cm^−1^ was due to OH bending, out-of-plane CH deformation and out-of-plane NH rocking, while the peak at 654 cm^−1^ corresponded to out-of-plane vibration of OH, NH, and CH. Finally, the vibration at 600 cm^−1^ was due to the formation of chemical interactions of nickel with the OH groups of chitosan [58].

The calculated FTIR spectrum for chitosan hydrogel (Figure 2d) confirms the presence of the polysaccharide’s characteristic functional groups. Stretch signals from the glycosidic backbone (C-O-C) are observed around 1000–1100 cm^−1^, as well as aliphatic CH vibrations near 2900 cm^−1^. More importantly, the spectrum clearly shows peaks corresponding to free amino and hydroxyl groups in the 1500–1600 cm^−1^ and 3200–3600 cm^−1^ regions, respectively. These functional groups (OH and NH) act as the main active coordination sites, giving chitosan hydrogel high capacity for adsorbing heavy metals such as mercury, copper, and nickel [57,58].

The shift of the bands corresponding to the hydroxyl and amino groups after the adsorption process confirms the participation of these groups in metal chelation. The donation of unshared electron pairs from the oxygen and nitrogen atoms to the empty orbitals of the metal cations decreases the electron density of the bonds. This electron delocalization attenuates bond strength constant, requiring a lower vibrational transition energy, resulting in the shift of the absorption bands. The difference in shift for Hg^2+^, Cu^2+^, and Ni^2+^ is attributable to variations in the intrinsic charge density and electron affinity of each metal [59].

### 3.2. Hydrogel Characterization Before and After Adsorption

The swelling percentage (SR %) exhibits opposing trends depending on the presence of metal ions due to marked changes in the structural dynamics of the chitosan hydrogel network (see Table 2). On the one hand, the increase in SR % in the free hydrogel with rising temperature is due to an endothermic process where the higher kinetic energy facilitates the partial dissociation of intra- and intermolecular hydrogen bonds. This thermodynamic relaxation increases conformational entropy and expands the free volume of the polymer chains, promoting greater diffusion and retention of the solvent into the matrix [39]. In contrast, the incorporation of cations (Hg^2+^, Cu^2+^, and Ni^2+^) significantly decreases water retention because these act as dense centers of physical cross-linking. By coordinating through Lewis’s acid–base interactions with reactive functional groups (such as free amines and hydroxyls), metals anchor multiple adjacent polymer chains, severely restricting their mobility, stiffening the three-dimensional network, and sterically displacing water molecules that previously interacted at those active sites, thus causing the macroscopic contraction observed in complexes in response to thermal agitation [60].

**Figure 3:**
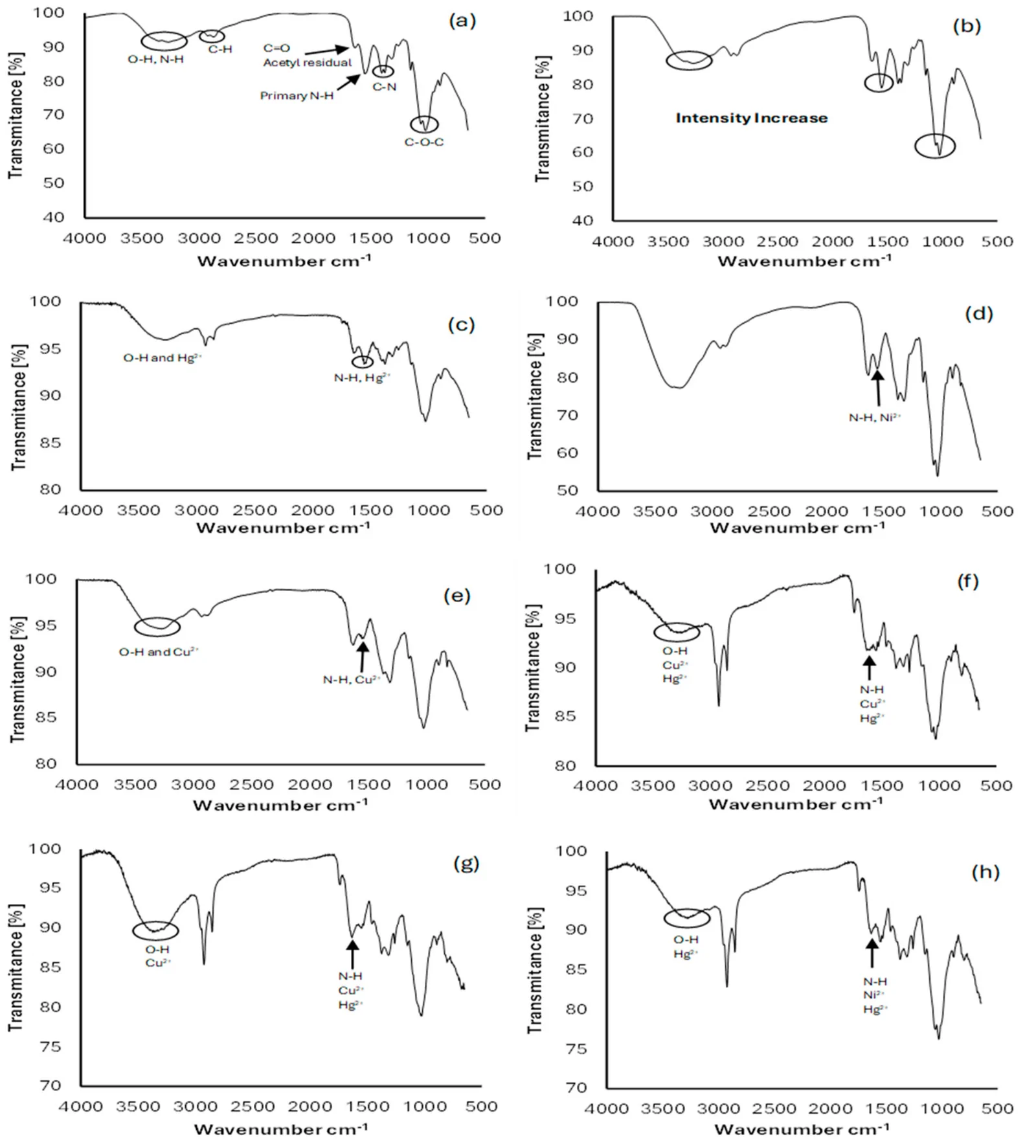
IR spectrum of (**a**) chitosan before cross-linking, (**b**) chitosan cross-linked with glutaraldehyde, (**c**) hydrogel loaded with Hg^2+^, (**d**) hydrogel loaded with Ni^2+^, (**e**) hydrogel loaded with Cu^2+^, (**f**) hydrogel loaded with Hg^2+^ and Cu^2+^, (**g**) hydrogel loaded with Ni^2+^ and Cu^2+^, and (**h**) hydrogel loaded with Hg^2+^ and Ni^2+^.

Figure 3a presents the FTIR of chitosan before cross-linking. In this spectrum, the regions of 3343 and 3258 cm^−1^ show the stretching of OH and NH, respectively, as well as hydrogen bonding. The peaks at 2919 and 2868 cm^−1^ corresponded to the symmetric and asymmetric stretching of CH. Between 1631 and 1316 cm^−1^, the vibrations were attributed to the residual acetyl groups, specifically to C=O and C-N. Evidence of primary amines (-NH_2_) was provided by the band at 1547 cm^−1^. The bands from 1403 to 1375 cm^−1^ were assigned to CH_2_ and CH_3_ groups, specifically rocking or symmetric bending. The prominent absorption in the 1036–1023 cm^−1^ range confirmed the stretching of C-O bonds, while the weak peak at 894 cm^−1^ was ascribed to the out-of-plane wagging of CH bonds in the monosaccharide units [61].

Figure 3b presents the FTIR spectrum of chitosan cross-linked with glutaraldehyde. In contrast to chitosan before cross-linking, a change in peak intensity is observed, particularly in the peaks corresponding to N-H and O-H bond rocking, as well as primary amine rocking, indicating an increased density of functional groups per unit volume due to the cross-linking of two or more chitosan chains with glutaraldehyde [62].

Figure 3c shows the IR corresponding to the hydrogel after the adsorption experiments with Hg. The peaks corresponding to the OH and NH groups at 3350–3250 and 1547 cm^−1^ decreased in intensity after Hg^2+^ adsorption; this may be due to the functional group participating in the formation of complexes with Hg^2+^.

Figure 3d reveals a notable change in the IR spectrum of the nickel adsorption at 1547 cm^−1^, which corresponded to only the primary amine groups participating in metal binding on the adsorbent surface, which likely contributes to the observed low adsorption capacity.

Figure 3e shows the IR of the hydrogel after copper adsorption. As with the binding of Hg^2+^, the corresponding peaks of amino and hydroxyl groups decreased, which corresponded to the binding of Cu^2+^ on these groups.

Figure 3f–h reveals a shift in the intensity of peaks associated with hydroxyl and amino functional groups, which indicates that, as in monocomponent systems, the OH and NH groups are the binding sites of heavy metals in binary solutions [63,64,65].

As in the theoretical analysis, the experimental FTIR shows a slight shift, although not as marked as in the theoretical IR. The shift of the O-H and N-H bands to lower wavenumbers is the physical evidence of chelation. By donating their electrons to the metal to form the complex, the original covalent bonds (O-H and N-H) lose electron density and weaken. A weaker bond requires less energy to vibrate, which is reflected in the spectrum as a shift [39].

Although the amino groups of the hydrogel are predominantly protonated as -NH_3_^+^ at pH = 4 (due to the typical pK_a_ ≈ 6.3–6.5 of chitosan), metal uptake still proceeds via a coordination-driven mechanism. The high binding affinity and formation constant of transition metals (Hg^2+^, Ni^2+^, and Cu^2+^) promote metal-induced deprotonation, shifting the protonation equilibrium (-NH_3_^+^ ⇆ -NH_2_ + H) and liberating Lewis-basic amine sites for coordination [55]. Furthermore, metal retention is assisted by synergistic coordination with adjacent hydroxyl groups (OH) and secondary ion-exchange phenomena, confirming that adsorption at slightly acidic conditions involves a cooperative chelation-complexation process [66].

**Figure 4:**
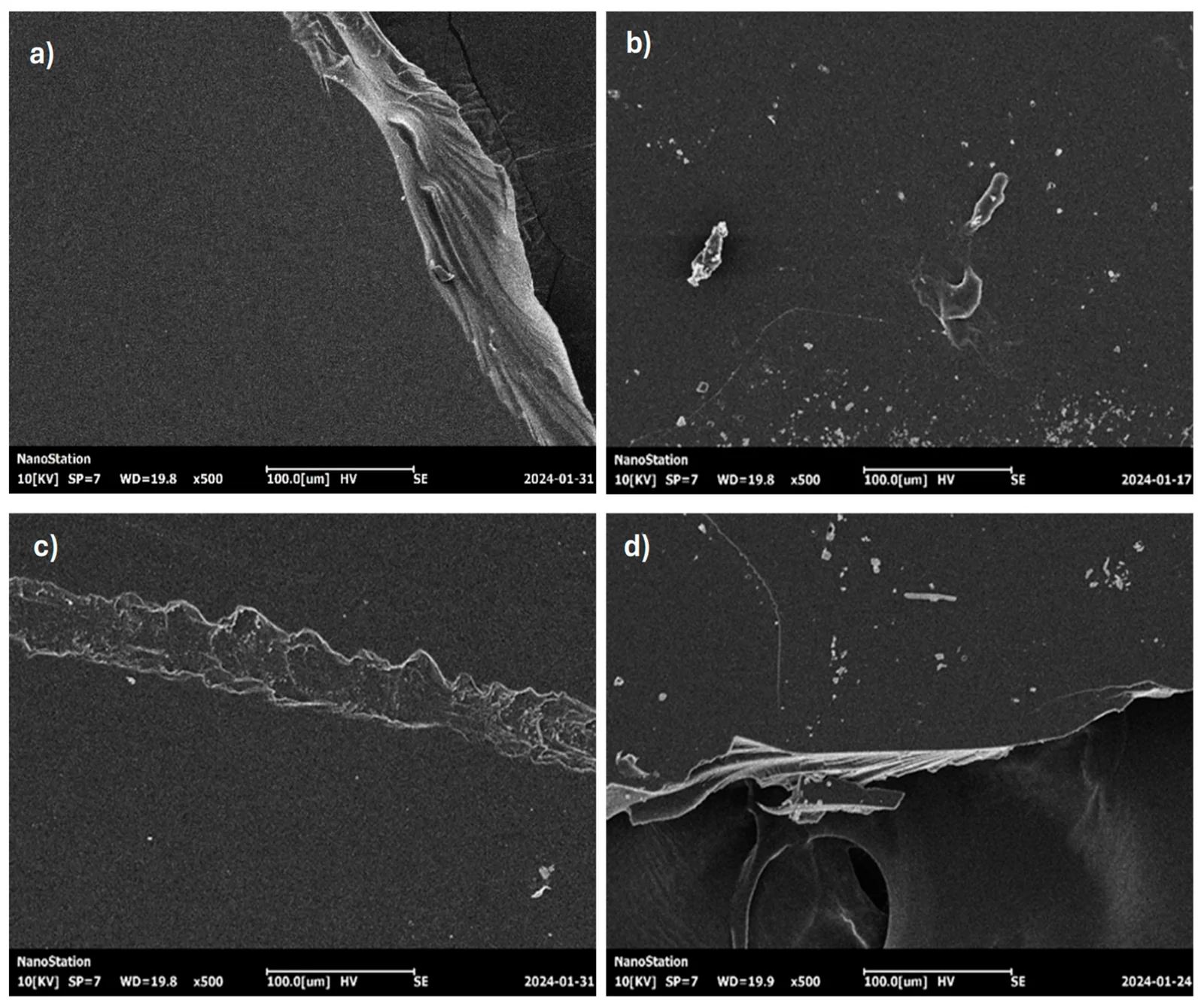
SEM images of chitosan/glutaraldehyde hydrogel (**a**) before the adsorption experiments, (**b**) hydrogel loaded with Hg^2+^, (**c**) hydrogel loaded with Ni^2+^, and (**d**) hydrogel loaded with Cu^2+^.

The surface morphology of the cross-linked chitosan hydrogel before and after heavy metal adsorption was evaluated by SEM (Figure 4). The pristine hydrogel (Figure 4a) shows a continuous, dense, and relatively smooth surface architecture devoid of macropores, accompanied by irregular wrinkles and morphological folds. This compact, non-porous skin layer is characteristic of xerogel matrices prepared via ambient evaporative drying; wherein capillary forces drive chain rearrangement and dense glutaraldehyde cross-linking via Schiff-base linkages during film solidification. Following the uptake of Hg^2+^, Ni^2+^, and Cu^2+^ (Figure 4b–d), a clear morphological transition is observed across the polymeric plane. The initially homogeneous surface develops fine, granular micro-domains and dispersed metallic clusters anchored across the hydrogel boundary. This noticeable increase in surface roughness confirms successful coordination and surface-complex entrapment of the metal cations onto the available Lewis-basic active sites of the functionalized network [67].

**Figure 5:**
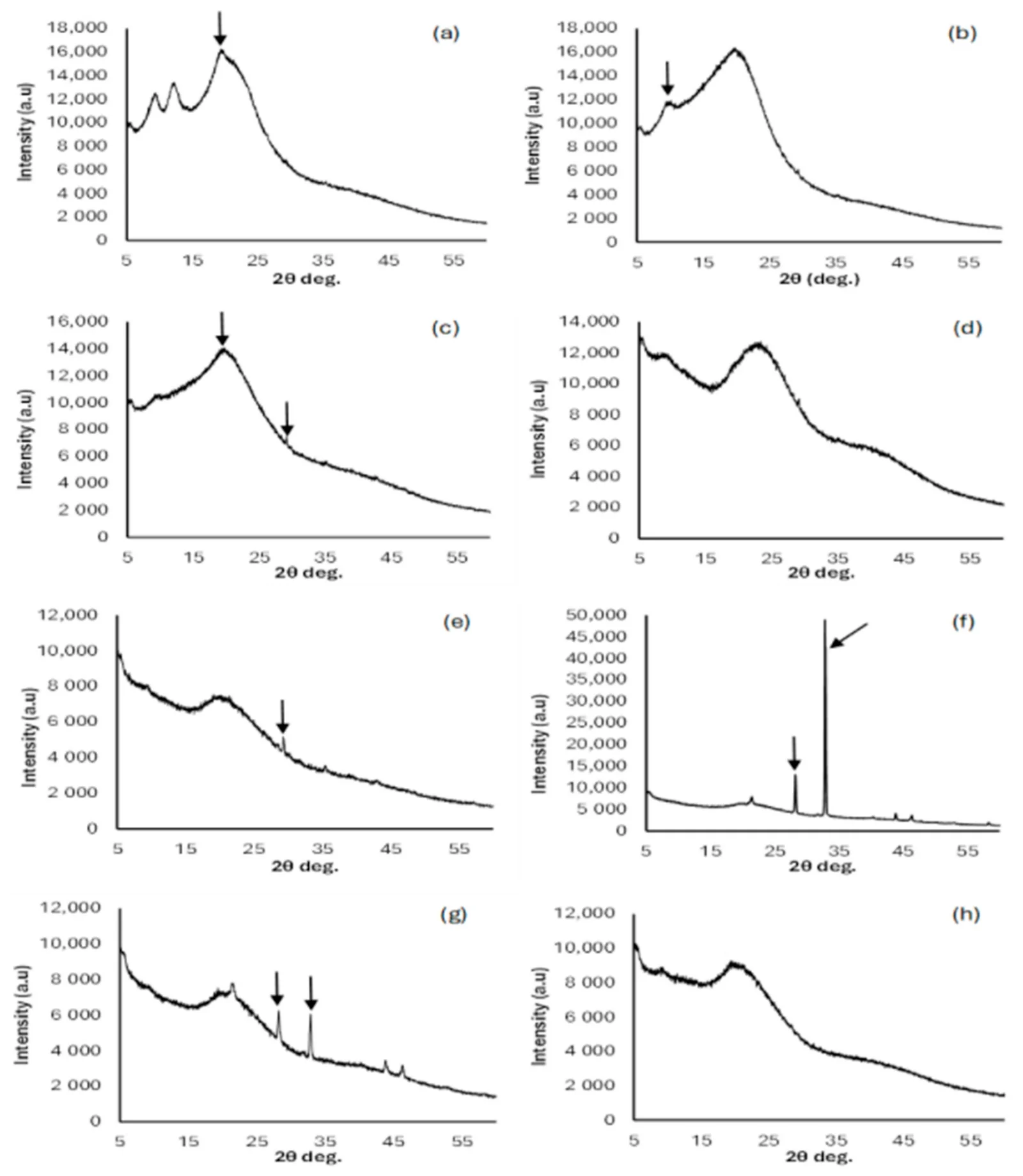
XRD of (**a**) chitosan before cross-linking, (**b**) chitosan cross-linked with glutaraldehyde, (**c**) hydrogel loaded with Hg^2+^, (**d**) hydrogel loaded with Ni^2+^, (**e**) hydrogel loaded with Cu^2+^, (**f**) hydrogel loaded with Hg^2+^ and Ni^2+^, (**g**) hydrogel loaded with Hg^2+^ and Cu^2+^, and (**h**) hydrogel loaded with Cu^2+^ and Ni^2+^.

Figure 5a shows the XRD of pure chitosan. This showed three characteristic diffraction peaks on the 2θ scale at 10°, 13°, and 20°, indicating high crystallinity due to the ordered structure of chitosan [68,69].

Figure 5b shows the diffractogram of the chitosan after cross-linking, where the peak at 13° disappeared and the peak at 10° decreased in intensity; that is, an amorphous structure was generated. This alteration was ascribed to the disruption of hydrogen bonds within the chitosan structure, resulting from the substitution of amino groups with imine groups, thereby perturbing the regular packing arrangement of the chitosan chains [70]. In the XRD patterns of the adsorbent after adsorption (see Figure 5c–h), an increase in crystallinity of the material was shown, specifically at 23°, 28°, 30°, 33°, 44°, and 46° attributed to the heavy metal ions on the material, specifically mercury chloride, nickel nitrate, and copper oxide.

In Figure 5c, we observe the characteristic broad peak of the amorphous polymer matrix near 2θ = 19–20° (left arrow). However, a small, sharp peak emerges around 2θ = 29° (right arrow). This suggests that the coordination of mercury with the active sites induces slight formation of localized crystalline domains or microprecipitation of the metal within the hydrogel network.

In Figure 5d, the amorphous nature is completely dominant. A broadening and possible displacement of the polymeric halo (2θ = 20–23°) is highlighted. Unlike mercury, nickel coordinates with the amine and hydroxyl groups, forming an entirely amorphous complex without inducing long-range spatial ordering in the chitosan chains.

Figure 5e shows that the behavior is analogous to that of mercury, but less pronounced. The amorphous phase predominates (2θ = 20°), but a very faint peak is detected near 2θ = 29° (arrow). Copper chelation generates a minimal degree of crystallinity, probably due to the specific geometry of the Cu-amine complex, which slightly stabilizes the local structure.

In Figure 5f, the diffractogram reveals a pronounced structural transition upon Hg/Ni co-adsorption, characterized by extremely sharp, high-intensity crystalline reflections emerging at 2θ ≈ 28° and 33°, along with secondary signals around 45–48°. While individual Ni^2+^ adsorption maintains an essentially amorphous polymeric profile and Hg^2+^ displays only weak localized reflection, the simultaneous presence of both cations under competitive conditions promotes the localized co-precipitation of inorganic crystalline phases (predominantly basic mercury/nickel salts or oxychloride/hydroxy-nitrate micro-crystallites formed at the localized interfacial double layer). The appearance of these defined narrow peaks is thus attributed to the nucleation of segregated inorganic crystalline micro-domains on the hydrogel surface rather than structural ordering of the polymeric chains, which fundamentally remain amorphous.

An analogous diffraction profile is observed in Figure 5g for the Hg/Cu binary system, displaying identical reflections at 2θ ≈ 28° and 33°, albeit with lower relative intensity. This indicates that the competitive presence of Cu^2+^ similarly triggers interfacial micro-precipitation in the presence of mercury, whereas the Cu/Ni system (Figure 5h) retains a purely amorphous halo (2θ ≈ 20°). This comparative behavior confirms that Hg^2+^, owing to its high atomic scattering factor and strong tendency toward mineral phase nucleation, is the indispensable agent promoting the segregated inorganic crystalline domains observed in the multi-metallic hydrogels [59,71].

### 3.3. Single and Binary Adsorption

#### 3.3.1. Kinetic Behavior

**Figure 6:**
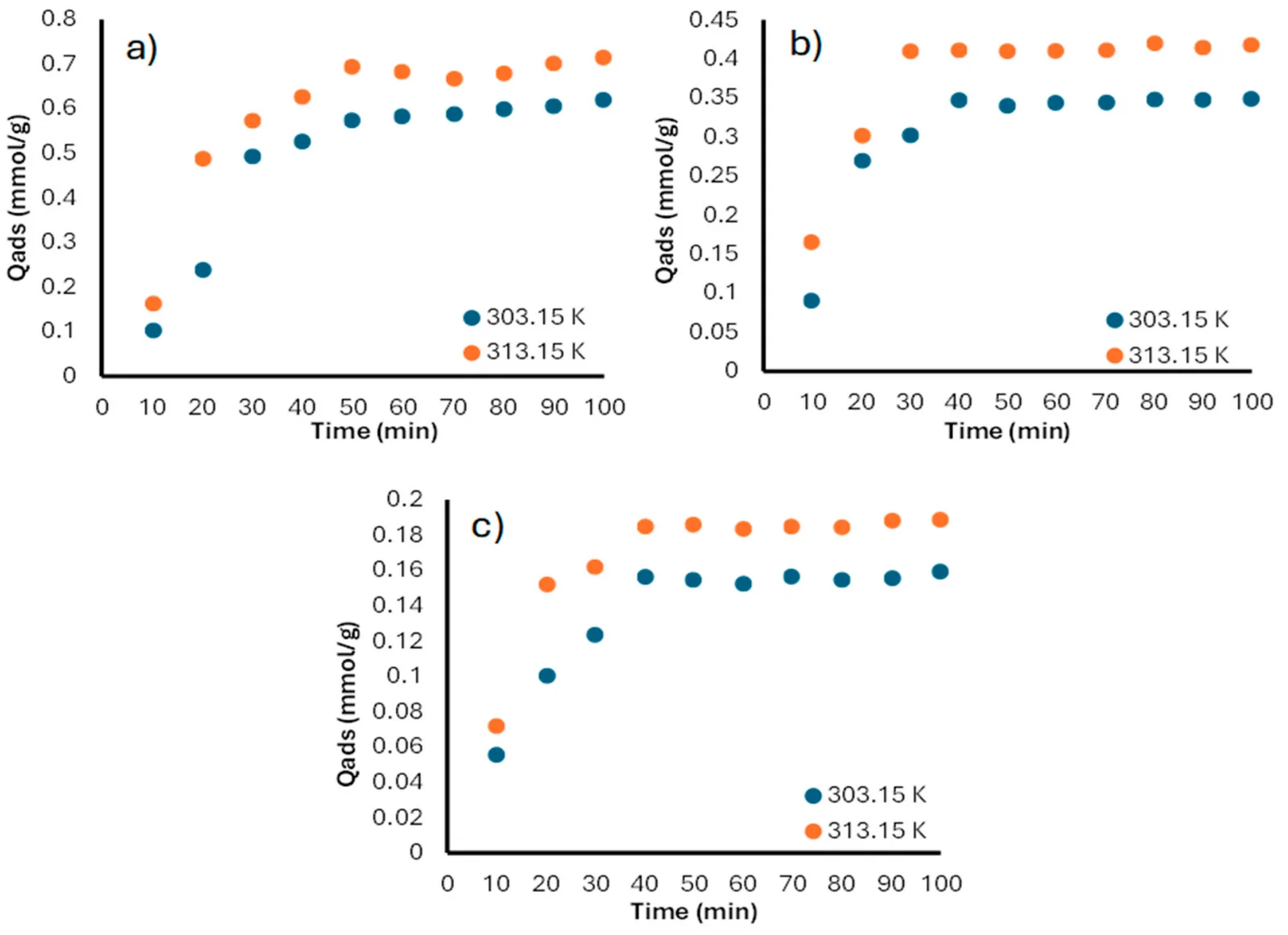
Experimental kinetics of (**a**) hydrogel–Hg^2+^, (**b**) hydrogel–Cu^2+^, and (**c**) hydrogel–Ni^2+^.

Experimental adsorption kinetics reveal that the removal of Hg^2+^, Cu^2+^, and Ni^2+^ by the hydrogel is a temperature-dependent process (Figure 6). In all cases, increasing the temperature from 303.15 K to 313.15 K resulted in an increase in Q_ads_, suggesting an endothermic nature to the adsorption process. The material demonstrated a higher affinity for Hg^2+^, achieving superior adsorption capacities compared to Cu^2+^ and Ni^2+^. Furthermore, saturation of the active sites occurs rapidly for copper and nickel (around 40 min), while mercury, despite achieving greater retention, requires a slightly longer time (approximately 60 min) to reach kinetic equilibrium.

**Figure 7:**
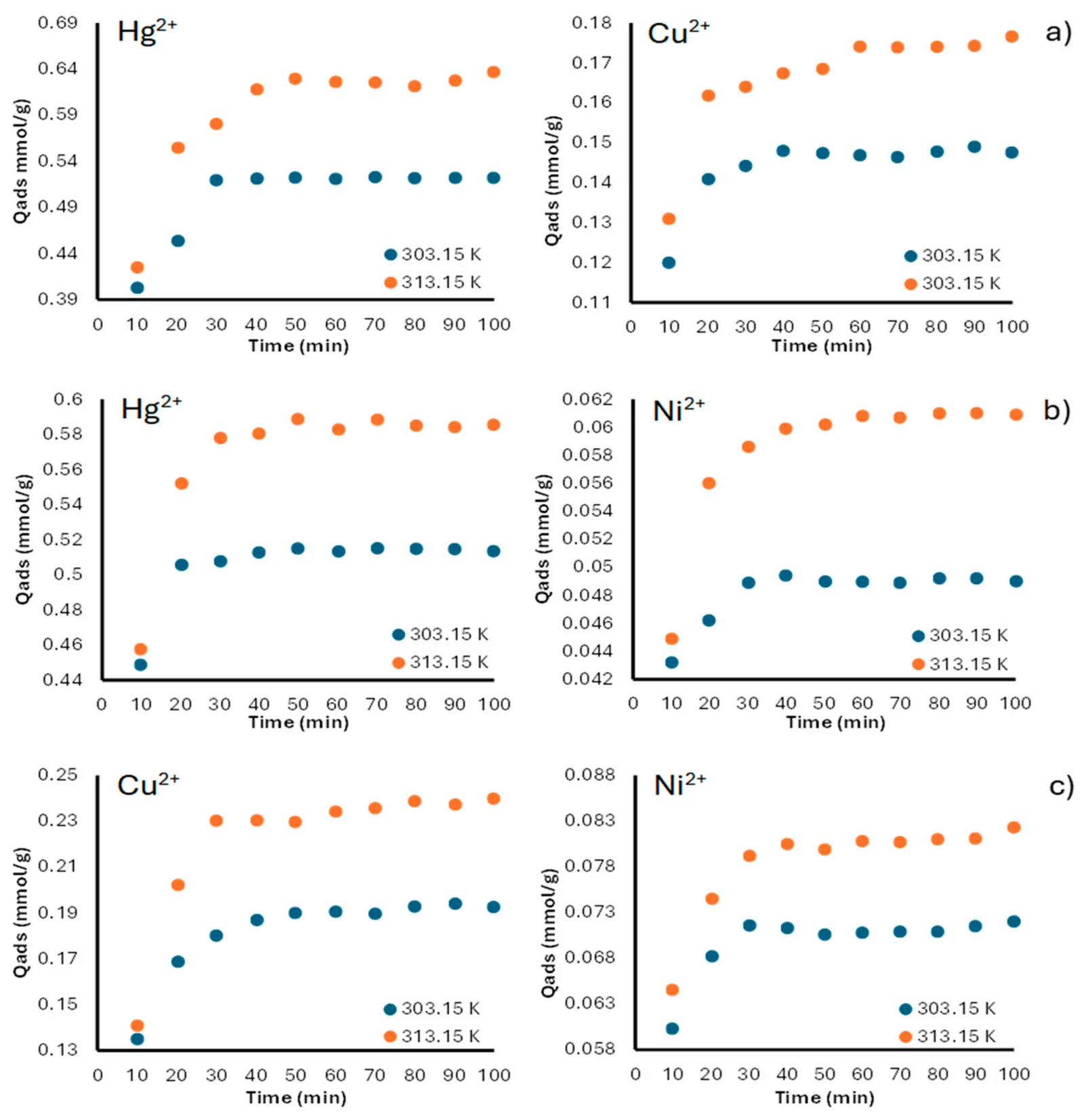
Binary experimental kinetics of (**a**) Hg^2+^–Cu^2+^, (**b**) Hg^2+^–Ni^2+^, and (**c**) Cu^2+^–Ni^2+^.

The kinetics of the binary systems show an accelerated initial adsorption rate during the first 20 to 30 min, attributed to the high availability and rapid occupancy of the hydrogel’s surface-active sites (Figure 7). Under bimetallic competition conditions, the times to reach dynamic equilibrium are comparatively short and are established simultaneously for both ions in the mixture, with the curves stabilizing between 40 and 60 min. Notably, Ni^2+^, being the cation with the lowest affinity in its respective mixtures, experiences rapid saturation of its available sites, reaching a premature plateau (approximately at 30–40 min). Finally, increasing the temperature to 313.15 K does not affect the equilibration time; that is, the competition kinetics and diffusion toward the active sites remain rapid despite the increase in the total amount adsorbed.

To elucidate the rate-limiting mechanism of adsorption, experimental data from single-component systems were evaluated using pseudo-first- and pseudo-second-order models. Table 3 shows that the pseudo-second-order model provides the best fit for the three metal ions at both temperatures, the determination coefficients (R^2^) were consistently above 0.995. This behavior suggests that the adsorption process in the hydrogel is predominantly controlled by chemisorption, which involves valence forces through the exchange or sharing of electrons between the active sites and the metals.

On the other hand, the analysis of the pseudo-second-order rate constants (k_2_) reveals an order of magnitude of Ni^2+^ > Cu^2+^ > Hg^2+^, which is inversely proportional to the adsorption affinity. This indicates that the saturation of available sites for Ni^2+^ occurs considerably faster compared to Hg^2+^, which is consistent with the observed time profiles. Furthermore, a decrease in k_2_ values is observed as the temperature increases to 313.15 K, a phenomenon attributable to the rapid initial surface saturation, which increases diffusion resistance in later stages of the process [42].

The kinetic parameters of the binary systems (Table 4) confirm that the pseudo-second-order model maintains the best linear fit (R^2^ > 0.997) compared to the pseudo-first-order model. This indicates that, even under competitive conditions, the dominant adsorption mechanism remains chemisorption. The behavior of the rate constants (k_2_) is of particular interest; the cations with lower affinity in their respective mixtures (for example, Ni^2+^ in the presence of Hg^2+^) exhibit exceptionally high k_2_ values (up to 47.00 min^−1^ at 303.15 K). This kinetic acceleration corroborates that the less favored ion almost instantaneously saturates the minority fraction of sites it manages to occupy, being rapidly displaced or blocked by the cation with higher affinity (Hg^2+^), which progressively dominates the material’s surface. Furthermore, the consistent increase in the calculated values of q_e_ to 313.15 K reaffirms the endothermic nature of the competitive retention process [72].

The apparent pseudo-second-order rate constants (K_2_) exhibit a downward trend as temperature increases from 303.15 K to 313.15 K (e.g., decreasing from 2.2005 to 1.9087 for Hg^2+^, from 5.1976 to 2.8469 for Cu^2+^, and from 7.4940 to 5.7886 for Ni^2+^). While an elementary rate constant for an endothermic chemisorption process is expected to follow Arrhenius behavior and increase upon heating, K_2_ operates as an apparent lumped parameter intrinsically linked to the equilibrium capacity through the initial sorption rate (h = k_2q_^2^_e_). In endothermic systems where q_e_ increases markedly with temperature, the mathematical formulation of K_2_ often decreases due to the prominent quadratic q_e_^2^ denominator [72,73].

More fundamentally, this decrease signifies a transition in the rate-limiting step from early interfacial coordination toward intraparticle diffusion control at 313.15 K. At this temperature, significant hydrogel swelling (reaching 178.57%) expands the macromolecular network; while this conformational relaxation unmasks deeper functional groups, it concurrently elongates internal diffusion pathways and elevates steric and boundary layer resistance. This is consistent with the Weber–Morris multi-stage analysis, confirming that the overall uptake is co-governed by surface coordination and strong internal mass-transfer resistance during the later stages.

The kinetic mass transfer mechanism of Hg^2+^, Cu^2+^, and Ni^2+^ across both single and binary systems was elucidated using the Weber–Morris intraparticle diffusion model (q_t_ = *kt*^1/2^ + C), as summarized in Table 5. The data clearly resolves into distinct multi-stage mass transport regimes for all studied systems. In the single-component systems, the initial period (t ≤ 40 min) is governed by substantially higher rate constants (*k*_1_ > *k*_2_), following the affinity sequence Hg^2+^ > Cu^2+^ > Ni^2+^ (e.g., 0.1454, 0.0824, and 0.0342 mmol/g min^1/2^ at 313.15 K, respectively). This primary step represents rapid liquid film mass transfer and ion penetration into the outer swollen macroporous domains of the polymeric network (178% swelling degree).

In contrast, the subsequent stage (40–100 min) exhibits a marked decline in diffusion rates (*k*_2_), driven by the depletion of available chelating sites and increased steric resistance within the dense cross-linked core. The non-zero intercept constants (*C*_2_ > 0), reaching up to 0.5458 mmol/g for Hg^2+^ at 313.15 K) confirm that while intraparticle pore diffusion controls the later uptake period, it is not the sole rate-limiting mechanism, operating in combination with boundary layer resistance [74,75]. For the binary competitive systems (Hg–Cu, Hg–Ni, and Cu–Ni), a noticeable reduction in $k_{1}$ values and an increase in boundary layer thickness (*C*_1_ > 1) were obtained relative to the individual systems. This kinetic deceleration is attributed to competitive site blocking, boundary layer expansion, and mutual steric hindrance at the pore apertures, with Hg^2+^ consistently maintaining superior mass-transfer rates and site occupancy over Cu^2+^ and Ni^2+^.

#### 3.3.2. Adsorption Behavior

**Figure 8:**
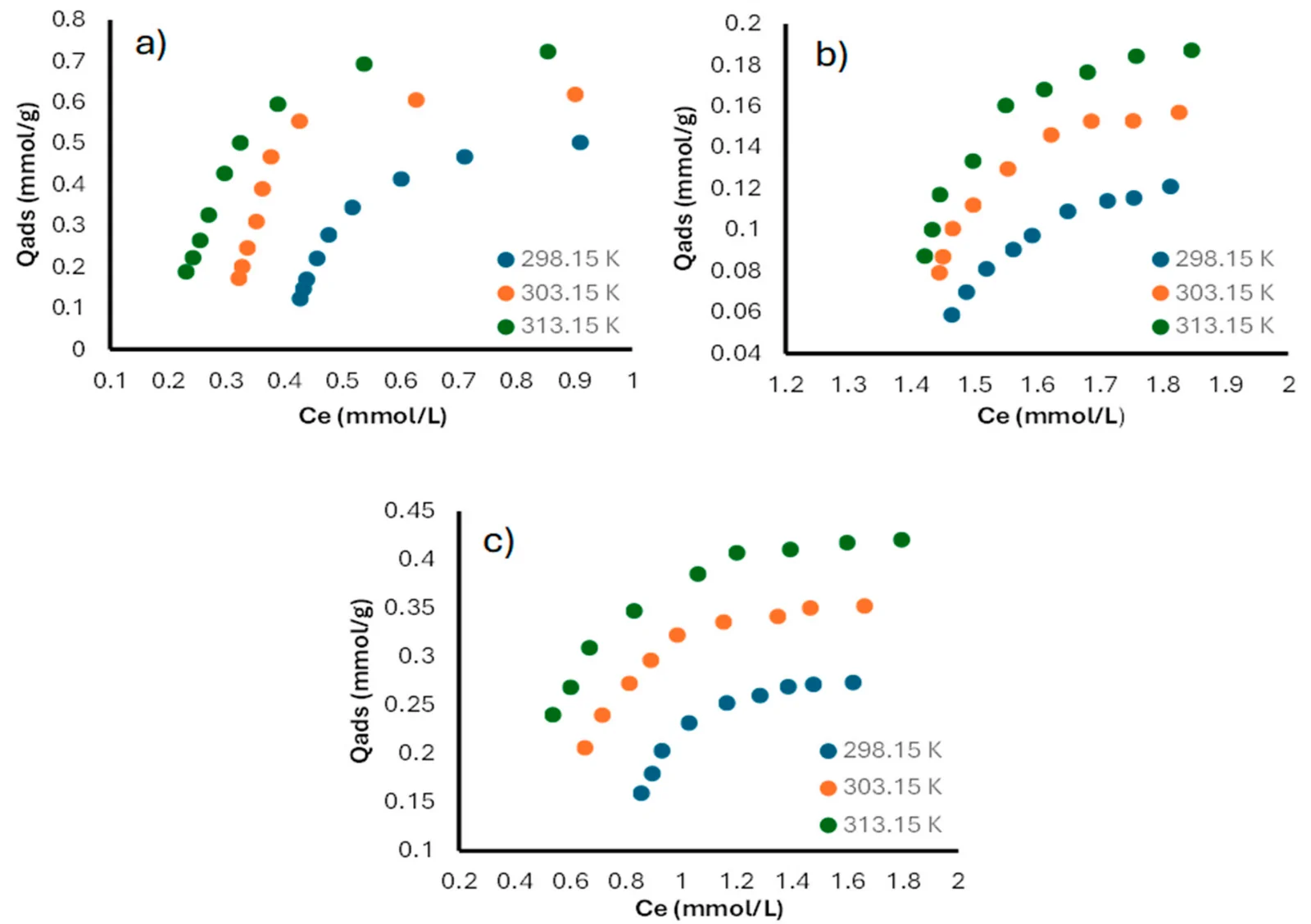
Single adsorption isotherms of (**a**) Hg^2+^, (**b**) Ni^2+^, and (**c**) Cu^2+^ on chitosan hydrogel at pH 4 and 298–313.15 K. Initial metal concentration of 2 mmol/L.

Figure 8 shows the experimental isotherms from single adsorption of heavy metals ions on chitosan-based adsorbent. Figure 8a shows the isotherm of Hg^2+^, with the maximum adsorption capacity of this metal reaching 0.723 mmol/g. For Cu^2+^ (Figure 8b), the maximum adsorption capacity was 0.420 mmol/g, while, for Ni^2+^ (Figure 8c), it was 0.187 mmol/g, all at 313.15 K. The isotherms corresponded to a favorable sorption process, exhibiting the typical Langmuir form with a strict plateau [76]. Isotherms indicated an endothermic process where the adsorption of all adsorbates increased with temperature. The increments in temperature could decrease the solution viscosity and improve the diffusion rate of Hg^2+^, Ni^2+^, and Cu^2+^ ions on the active sites (i.e., functional groups) of the adsorbent.

**Figure 9:**
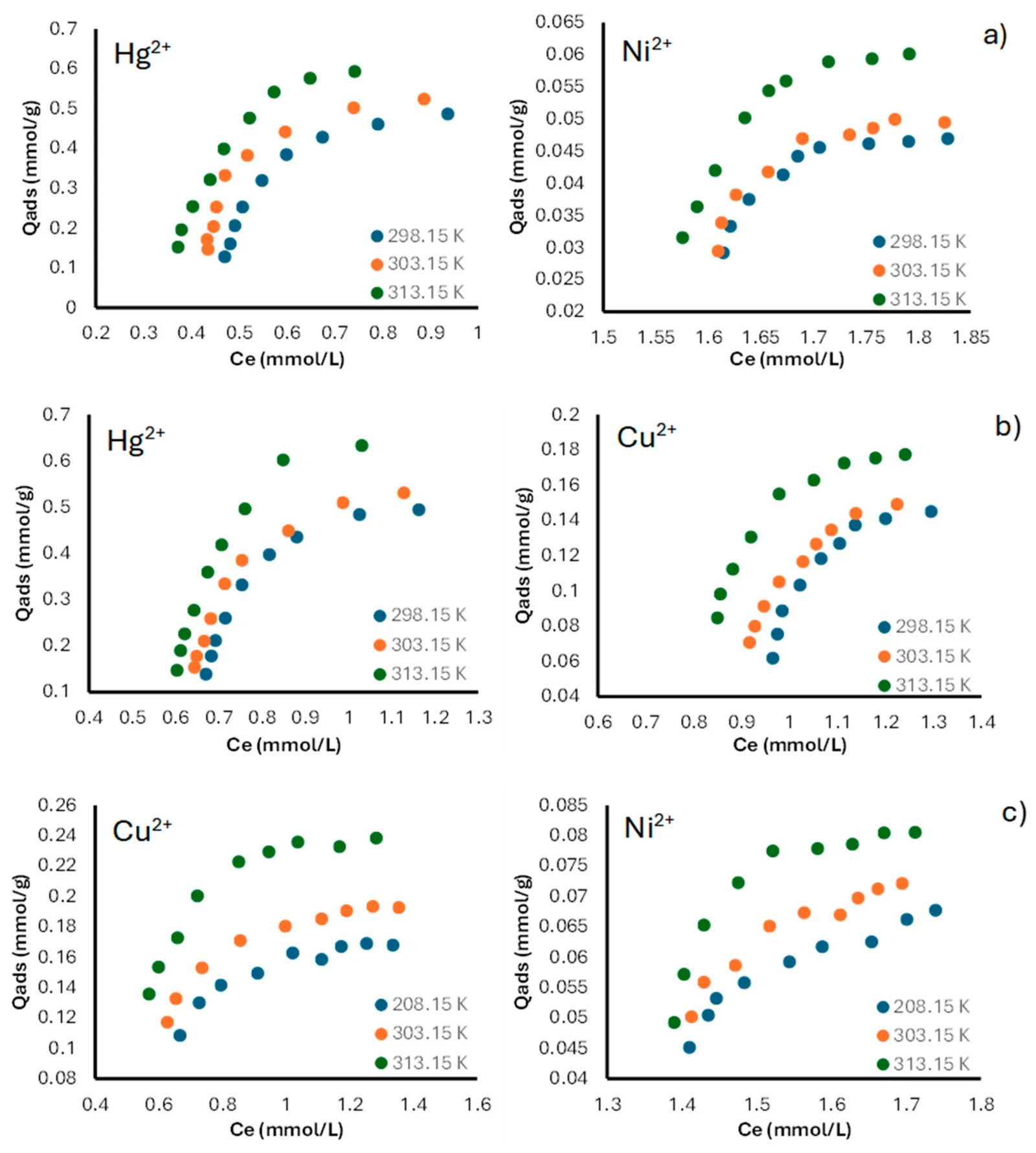
Binary adsorption isotherms of (**a**) Hg^2+^–Ni^2+^, (**b**) Hg^2+^–Cu^2+^, and (**c**) Cu^2+^–Ni^2+^ on chitosan hydrogel at pH 4 and 298–313.15 K. Initial metal concentration of 2 mmol/L.

Figure 9 shows the experimental isotherms obtained from the binary adsorption of the heavy metals on hydrogels. In Figure 9a, the isotherms of the Hg^2+^ and Ni^2+^ mixture are shown; the maximum adsorption capacities were 0.593 and 0.060 mmol/g, respectively, at 313.15 K. The isotherms of Hg^2+^ and Cu^2+^ in binary solution are shown in Figure 9b. The adsorption capacities were 0.633 and 0.177 mmol/g, respectively, at a temperature of 313.15 K. Finally, in Figure 9c, the mixture of Cu^2+^ and Ni^2+^ is shown, with the maximum adsorption capacities achieved in this system reaching 0.238 and 0.080 mmol/g, respectively, at the same temperature. However, the maximum adsorption capacity for all heavy metal ions was compromised, with decreases of 17.98%, 57.85%, and 67.91% for Hg^2+^, Cu^2+^, and Ni^2+^, respectively. This reduction was attributed to antagonistic adsorption, where heavy metal ions competed for the hydrogel’s active sites [77]. The reusability and operational stability of the hydrogel were evaluated over five consecutive adsorption–desorption cycles under identical conditions (T = 40 °C, pH = 4) (see Table 6).

A gradual decrease in adsorption performance was observed for all three metal ions across successive cycles. For Hg^2+^, the adsorbent retained 90.3% and 72.2% of its original capacity during cycles 2 and 3, respectively, before experiencing a more pronounced drop in cycles 4 (38.9%) and 5 (18.1%). In contrast, Cu^2+^ and Ni^2+^ exhibited faster decay rates, retaining only 47.6% and 38.9% by cycle 3, and dropping to <12% by the fifth cycle. This continuous decrease in retention capacity can be attributed to the incomplete stripping of strongly bound metallic complexes from the deeper micro- and mesoporous network, causing steric hindrance and occupation of chelating sites, and cumulative mechanical shrinkage of the polymeric matrix resulting from repeated swelling–desorption cycles at 40 °C.

Table 7 shows the fitting parameters of the Sips model for single-component and binary adsorption systems at different temperatures (298.15–313.15 K). The experimental data were successfully modeled using the Sips isotherm, yielding correlation coefficients greater than 0.96 (R^2^ > 0.96).

This fit, along with heterogeneity parameter values (n) less than one, confirms the high energy distribution and heterogeneity of the active sites present in the hydrogel network. The analysis at different temperatures (298.15–313.15 K) revealed the endothermic nature of the process, evidenced by the simultaneous increase in the maximum adsorption capacity (q_e_) and the affinity constant (K_s_) with increasing temperature, suggesting greater exposure of the anchoring sites due to thermal relaxation of the polymer matrix. Furthermore, the material demonstrated marked selectivity in the order Hg^2+^ > Cu^2+^ > Ni^2+^ for single-component systems, indicating a preferential chemical interaction toward mercury that is consistent with the high affinity of nitrogen functional groups for softer metals.

In Table 8, the fit to the Extended Sips model (R^2^ > 0.96) confirms that adsorption occurs on a heterogeneous surface, typical of polymer networks. The process exhibits an endothermic nature, evidenced by the universal increase in adsorption capacity (q_e_) when the temperature is raised from 298.15 to 313.15 K. Furthermore, the data reveals a marked selectivity hierarchy (Hg^2+^ > Cu^2+^ > Ni^2+^).

**Figure 10:**
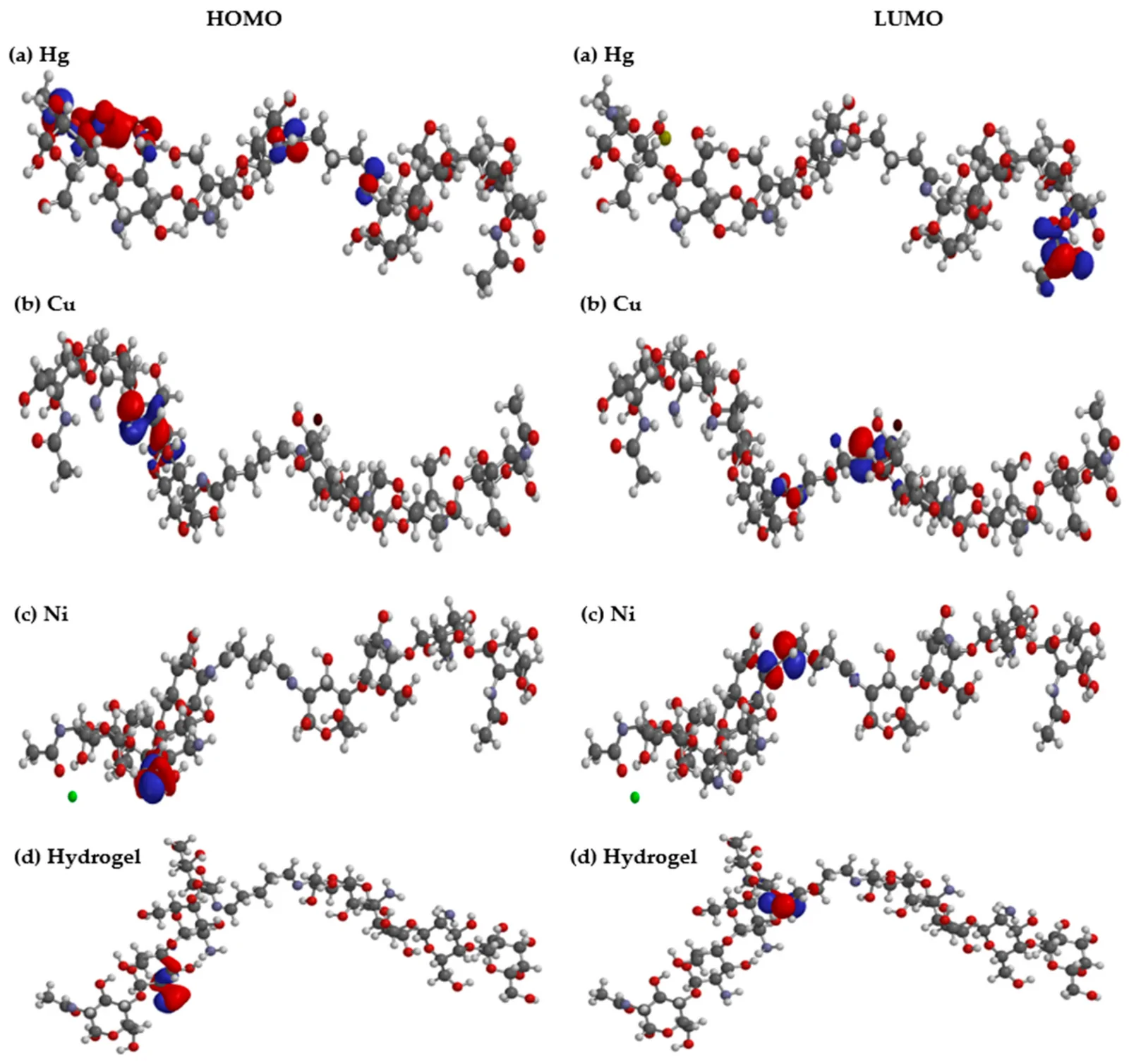
Molecular orbitals of the adsorption of (**a**) mercury, (**b**) copper, (**c**) nickel, and (**d**) hydrogel. Red color: oxygen, white color: hydrogen, yellow color: mercury, brown color: copper, blue color: nitrogen, gray color: carbon, and green color: nickel, respectively.

On the other hand, Table 9 shows the enthalpy changes using the van’t Hoff equation, which indicates that the adsorption processes were carried out by chemisorption [77], which agrees with the band gap determined for the molecular orbitals (see Figure 10) in the Spartan ‘14 software using DFT. Adsorption enthalpies (ΔH° > 40 kJ/mol) indicate chemisorption [78], which is corroborated in the FTIR spectra by the band shifts of the NH and OH groups, demonstrating the formation of coordination bonds. Additionally, the ΔG° and ΔS° parameters confirm the thermodynamic spontaneity of the process and the entropic increase resulting from ionic dehydration during anchoring to the polymer network. The hydrogel/Ni^2+^ complex exhibited the largest band gap (2.39 eV), indicating the formation of a chemically harder system with less electron delocalization in the coordination bond compared to mercury.

This greater resistance to electron transfer explains why Ni^2+^ adsorption releases the least amount of energy (ΔH° = 40.94 kJ/mol), placing it just at the lower limit that defines chemisorption, while softer, more polarizable ions such as Hg^2+^ show reduced band gaps and significantly higher enthalpies [79].

Table 10 shows the adsorption capacities for Hg^2+^, Ni^2+^, and Cu^2+^ in both single-component and binary systems. The results fall within the ranges previously reported in the literature, indicating that the system behavior is consistent with that expected for functionalized polymeric and hybrid adsorbent materials for heavy metal removal, particularly those based on chitosan. However, variations in adsorption capacity are observed in binary mixtures, suggesting the presence of competitive effects and possible selectivity phenomena among the metal ions, depending on their physicochemical properties and their interaction with the active sites of the adsorbent.

## 4. Conclusions

Chitosan-based hydrogels emerge as a competitive alternative for adsorption processes, offering significant potential for water treatment and purification applications. In this regard, the adsorption behavior of the three metals was spontaneous and endothermic, and increasing the temperature to 313.15 K enhanced the maximum adsorption capacity, confirming temperature as a key parameter. Furthermore, the consistent fitting of both single and binary systems to a pseudo-second-order model indicates that chemisorption governs the adsorption mechanism. Chitosan hydrogels allow the adsorption of metal ions due to amino and hydroxyl groups that provide active sites for chemisorption, and their high swelling ratio (up to 178.57%) facilitates mass transfer and accessibility of metal ions within the hydrogel film.

The physical and chemical properties of metal ions influence the adsorption mechanisms of metal ions; while Hg^2+^ has a greater interaction with OH groups, Ni^2+^ has a greater interaction with NH_2_ groups. Theoretical and experimental FTIR spectra corroborated that amino, and hydroxyl groups are responsible for the various adsorptions. Consistently, the swelling behavior showed a reduction after metal ion adsorption, suggesting that these interactions restrict network expansion and water uptake.

According to the results obtained, chitosan hydrogels are more effective at removing Hg^2+^ than Ni^2+^ and Cu^2+^, with the adsorption order being Hg^2+^ > Cu^2+^ > Ni^2+^. Scanning electron microscopy (SEM) confirmed the incorporation of metal ions within the hydrogel network, as evidenced by the observed morphological features. Regarding the X-ray diffraction analyses, single-component mixtures of the analyzed metal ions tend to induce structural disorder in the hydrogel. In contrast, binary combinations containing mercury induce the nucleation of localized inorganic crystalline micro-phases on the surface of the amorphous hydrogel network. The reusability tests revealed a significant decrease in adsorption performance from the third cycle onward, with the extent of deterioration depending on the metal ion, indicating that ion-specific interactions influence the hydrogel’s regeneration and reuse efficiency.

Computational simulations of chitosan-based hydrogels for metal ion adsorption constitute a complementary analytical approach to experimental studies, enabling a deeper understanding of interaction mechanisms while significantly reducing time and resource requirements. Careful selection of DFT parameters ensured reliable modeling of metal–hydrogel interactions and supported the experimental findings.

## Figures and Tables

**Table 1:** Properties of adsorption process.

Properties	Units	Hydrogel—Hg^2+^	Hydrogel—Cu^2+^	Hydrogel—Ni^2+^
ΔGads° (298.15 K)	kJ/mol	−15.1	−14.5	−5.29
ΔHads° (298.15 K)	kJ/mol	65.5	43.7	40.27
ΔGads° (303.15 K)	kJ/mol	−15.6	−15	−6.39
ΔHads° (303.15 K)	kJ/mol	68.7	44.9	40.91
ΔGads° (313.15 K)	kJ/mol	−18.5	−17.3	−7.33
ΔHads° (313.15 K)	kJ/mol	69.8	45.1	41.82
Dipole moment	Debye	26.20	29.70	23.14
Polarizability	Å^3^	36.82	41.63	40.63

**Table 2:** Percent swelling rate (SR %) of hydrogel at different temperatures.

Sample	298.15 K (SR %)	303.15 K (SR %)
Hydrogel	166.66	178.57
Hydrogel–Hg^2+^	109.43	102.44
Hydrogel–Cu^2+^	132.67	129.89
Hydrogel–Ni^2+^	153.12	150.23

**Table 3:** Kinetic parameters of single-component adsorption of heavy metals.

Kinetic Model		Hg^2+^		Cu^2+^		Ni^2+^	
	Kinetic Parameters	303.15 K	313.15 K	303.15 K	313.15 K	303.15 K	313.15 K
Pseudo-first order	q_e_ (mmol/g)	0.6118	0.7016	0.3447	0.4143	0.1550	0.1853
	K_1 (_min^−1^)	0.3064	0.2806	0.3162	0.2663	0.2754	0.2601
	R^2^	0.992	0.998	0.991	0.990	0.987	0.996
Pseudo-second order	q_e_ (g/(mg·min)	0.6217	0.7167	0.3486	0.4210	0.1577	0.1886
	K_2_ (g mg^−1^ min^−1^)	2.2005	1.9087	5.1976	2.8469	7.4940	5.7886
	R^2^	0.9950	0.9990	0.9990	0.9980	0.9980	0.9990

**Table 4:** Kinetic parameters of binary adsorption of heavy metals.

Adsorbate	T (K)	Pseudo-First Order			Pseudo-Second Order		
		q_e_ (mmol/g)	K_1_ (min^−1^)	R^2^	q_e_ (mmol/g)	K_2_ (g mg^−1^ min^−1^)	R^2^
Hg^2+^–Cu^2+^	303.15	0.5218	0.2891	0.999	0.5293	3.1274	0.998
	313.15	0.6242	0.2709	0.998	0.6356	1.7672	0.999
Cu^2+^–Hg^2+^	303.15	0.1478	0.2953	0.998	0.1496	10.7331	0.998
	313.15	0.1741	0.3172	0.997	0.1758	9.5815	0.998
Hg^2+^–Ni^2+^	303.15	0.5205	0.2494	0.998	0.5299	1.9786	0.997
	313.15	0.5836	0.3105	0.998	0.5894	3.3625	0.999
Ni^2+^–Hg^2+^	303.15	0.0491	0.3240	0.997	0.0495	47.0037	0.998
	313.15	0.0593	0.2823	0.997	0.0603	20.4580	0.998
Ni^2+^–Cu^2+^	303.15	0.0709	0.2666	0.997	0.0722	15.6542	0.999
	313.15	0.0797	0.3162	0.998	0.0808	19.607	0.999
Cu^2+^–Ni^2+^	303.15	0.1904	0.3253	0.998	0.1928	8.9798	0.998
	313.15	0.2339	0.2884	0.998	0.2374	5.7125	0.999

**Table 5:** Weber–Morris intraparticle diffusion model.

Adsorbate	T (K)	k_1_ (mmol/g min^1/2^)	C_1_ (mmol/g)	R^2^	k_2_ (mmol/g min^1/2^)	C_2_ (mmol/g)	R^2^
Hg^2+^	303.15	0.1458	−0.3685	0.9452	0.0214	0.4084	0.8852
Hg^2+^	313.15	0.1454	−0.2438	0.9048	0.0163	0.5458	0.5697
Cu^2+^	303.15	0.0787	−0.1303	0.9079	0.0014	0.3342	0.3263
Cu^2+^	313.15	0.0824	−0.0783	0.9312	0.0023	0.3942	0.5549
Ni^2+^	303.15	0.0310	−0.0416	0.9932	0.0007	0.1500	0.1826
Ni^2+^	313.15	0.0342	−0.0234	0.8971	0.001	0.1777	0.4069

**Table 6:** Reusability cycles for Hg^2+^, Cu^2+^, and Ni^2+^ adsorption.

Cycle	Hg^2+^		Cu^2+^		Ni^2+^	
	Q_ads_ (mmol/g)	RE (%)	Q_ads_ (mmol/g)	RE (%)	Q_ads_ (mmol/g)	RE (%)
1	0.72	100	0.42	100	0.18	100
2	0.65	90.3	0.29	69	0.11	61.1
3	0.52	72.2	0.20	47.6	0.07	38.9
4	0.28	38.9	0.12	28.6	0.03	16.7
5	0.13	18.1	0.05	11.9	0.01	0.6

**Table 7:** Sips model parameters of the adsorption process.

Adsorbate	T (K)	q_e_ (mmol/g)	Ks (L/mmol)	n	R^2^
Hg^2+^	298.15	0.4852	2.1275	0.1116	0.9878
	303.15	0.6105	2.8849	0.0803	0.9965
	313.15	0.7166	3.5712	0.1839	0.9979
Cu^2+^	298.15	0.2743	1.2282	0.1404	0.9967
	303.15	0.3569	1.6385	0.2316	0.9961
	313.15	0.4330	2.0053	0.3494	0.9960
Ni^2+^	298.15	0.1226	0.6823	0.0584	0.9948
	303.15	0.1567	0.6965	0.0465	0.9944
	313.15	0.1864	0.7037	0.0520	0.9872

**Table 8:** Extended Sips model parameters of the binary adsorption process.

Adsorbate	T (K)	q_e_ (mmol/g)	Ks_1_ (L/mmol)	Ks_2_ (L/mmol)	n_1_	n_2_	R^2^
Hg^2+^–Cu^2+^	298.15	0.4925	1.4046	1.2282	0.0843	0.1404	0.990
	303.15	0.5317	1.4588	1.6385	0.0881	0.2316	0.989
	313.15	0.6532	1.5100	2.0053	0.1001	0.3494	0.995
Hg^2+^–Ni^2+^	298.15	0.4804	1.9647	0.6823	0.0975	0.0584	0.983
	303.15	0.4882	2.1905	0.6965	0.0822	0.0465	0.970
	313.15	0.6221	2.343	0.7037	0.1446	0.052	0.996
Cu^2+^–Hg^2+^	298.15	0.1522	1.0319	1.2282	0.0618	0.1116	0.984
	303.15	0.1594	1.0835	1.3385	0.0847	0.0803	0.994
	313.15	0.1735	1.181	1.8053	0.0762	0.1839	0.991
Cu^2+^–Ni^2+^	298.15	0.1746	1.7054	1.2282	0.2021	0.0584	0.979
	303.15	0.2049	1.7642	1.6385	0.2053	0.0465	0.995
	313.15	0.2597	1.8385	2.0053	0.1784	0.052	0.991
Ni^2+^–Cu^2+^	298.15	0.0597	0.7533	0.6823	0.0781	0.1404	0.968
	303.15	0.0746	0.7498	0.6965	0.0664	0.2316	0.971
	313.15	0.0853	0.7316	0.7037	0.0309	0.3494	0.989

**Table 9:** Thermodynamic parameters of the adsorption process.

Sample	T (°C)	T (K)	ΔG° (kJ/mol)	ΔH° (kJ/mol)	ΔS° (J/mol K)	Band Gap (eV)
	25	298.15	−14.63			1.05
Hydrogel/Hg^2+^	30	303.15	−16.06	70.70	286.20	
	40	313.15	−18.93			
	25	298.15	−14.04			2.04
Hydrogel/Cu^2+^	30	303.15	−15.03	44.74	197.14	
	40	313.15	−17.00			
	25	298.15	−5.38			2.39
Hydrogel/Ni^2+^	30	303.15	−6.15	40.94	155.36	
	40	313.15	−7.71			

**Table 10:** Adsorption capacities of Hg^2+^, Ni^2+^, and Cu^2+^ in mono- and binary systems.

Adsorbent	Adsorbate	q (mmol/g)	T (°C)	pH	Reference
**Monocomponent**					
Chitosan-Iron(III)	Hg^2+^	1.8	-	4.5	[80]
DNA–chitosan (DNA–CS) hydrogel	Hg^2+^	0.0498–0.0996	25	-	[81]
Xanthate-modified chitosan/poly(Nisopropylacrylamide)	Ni^2+^	1.1398	25	5.0	[82]
Alginate modified graphitic carbon nitride	Ni^2+^	5.22	20	6.0	[83]
Chitosan	Cu^2+^	0.243	-	5.25	[84]
Chitosan/orange peel	Cu^2+^	1.835	25	4.0, 5.0	[85]
Hydrogel based chitosan	Hg^2+^ Ni^2+^ Cu^2+^	0.187 0.723 0.420	40	4.0	This study
**Binary system**					
Chitosan-coated perlite beads Cu^2+^–Ni^2+^	Cu^2+^ Ni^2+^	2.3139 0.663	25	5.0	[86]
Chitosan/polyethyleneiminemagnetic hydrogels Cu^2+^–Ni^2+^	Cu^2+^ Ni^2+^	0.901 0.600	25	-	[43]
Alginate/polyethylenimine membranes Cu^2+^–Hg^2+^	Cu^2+^ Hg^2+^	0.931 0.650	20	6.0	[42]
Hydrogel based chitosan	Hg^2+^ Ni^2+^ Cu^2+^	0.633 0.080 0.238	40	4.0	This study

## Data Availability

The original contributions presented in this study are included in the article. Further inquiries can be directed at the corresponding author.
